# Global Call to Action to scale-up coverage of intermittent preventive treatment of malaria in pregnancy: seminar report

**DOI:** 10.1186/s12936-015-0730-3

**Published:** 2015-05-18

**Authors:** Koki Agarwal, Pedro Alonso, R Matthew Chico, Jane Coleman, Stephanie Dellicour, Jenny Hill, Maud Majeres-Lugand, Viviana Mangiaterra, Clara Menendez, Kate Mitchell, Elaine Roman, Elisa Sicuri, Harry Tagbor, Anna Maria van Eijk, Jayne Webster

**Affiliations:** Maternal and Child Health Integrated Program, Jhpiego, Baltimore, USA; Global Malaria Programme, World Health Organization, Geneva, Switzerland; Department of Disease Control, London School of Tropical Medicine and Hygiene, London, UK; Department of Clinical Sciences, Liverpool School of Tropical Medicine, Pembroke Place, Liverpool, UK; Access & Product Management, Medicine for Malaria Venture, Geneva, Switzerland; RMNCH and HSS Technical Advice & Partnerships Department, The Global Fund to Fight AIDS, Tuberculosis and Malaria, Vernier-Geneva, Switzerland; ISGlobal, Barcelona Centre for International Health Research (CRESIB), Hospital Clínic - Universitat de Barcelona, Barcelona, Spain; Maternal Health Task Force, Harvard School of Public Health, Boston, USA; Kwame Nkrumah University of Science and Technology, Department of Community Health, School of Medical Sciences, Kumasi, Ghana

**Keywords:** Malaria, Pregnancy, Intermittent preventive treatment, Call to action, Sub-Saharan Africa

## Abstract

In 2014, a global ‘Call to Action’ seminar for the scale-up of intermittent preventive treatment of malaria in pregnancy was held during the 63rd Annual Meeting of the American Society of Tropical Medicine and Hygiene. This report summarizes the presentations and main discussion points from the meeting.

**Electronic supplementary material:**

The online version of this article (doi:10.1186/s12936-015-0730-3) contains supplementary material, which is available to authorized users.

## Purpose of the meeting

On the 5th of November 2014, during the 63rd Annual Meeting of the American Society of Tropical Medicine and Hygiene in New Orleans, USA, the Roll Back Malaria (RBM) partnership Malaria in Pregnancy (MiP) Working Group and the Malaria in Pregnancy Consortium (MiPc), with support from Medicines for Malaria Venture and the London School of Hygiene and Tropical Medicine (LSHTM), hosted a global ‘Call to Action’ seminar for the scale-up of intermittent preventive treatment of malaria in pregnancy (IPTp) (see seminar agenda in Additional file 1). The meeting was attended by 80 representatives from 37 institutions across 15 countries, nine of which were sub-Saharan, including policymakers, programme managers, researchers, technical advisors and donors.

Administration of IPTp with sulphadoxine-pyrimethamine (SP) as part of routine antenatal care (ANC) is one of three key interventions recommended by the World Health Organization (WHO) for the control of malaria in pregnancy in areas of stable malaria transmission. The other two interventions are the provision of insecticide-treated nets and effective case management. Coverage has remained unacceptably low in countries with an IPTp policy. The aim of this seminar was to convene key stakeholders to review barriers to the scale-up of IPTp and to develop an action plan to increase coverage rapidly. This report summarizes central issues presented and discussed during the Call to Action seminar relating to the scale-up of IPTp. Key issues raised at the seminar have been used to outline a set of recommended actions as part of a Global Call to Action which aims to increase national and international coverage to rapid effect.

Pedro Alonso, the newly appointed Director of the WHO Global Malaria Programme (GMP), strongly voiced his support for increasing the coverage of IPTp and pledged that he would make the scale-up of IPTp a priority for GMP. Reinforcing this point, he said that until better and more cost-effective tools are available for the prevention of malaria in pregnancy, GMP will drive the scale-up for IPTp coverage.

*“Intermittent preventive therapy for pregnancy is a life-saving tool which will be a main priority for the WHO Global Malaria Programme team moving forward.”* (Pedro Alonso, Director of the Global Malaria Programme, World Health Organization)

### Status of IPTp coverage in sub-Saharan Africa

**Fig. 1:**
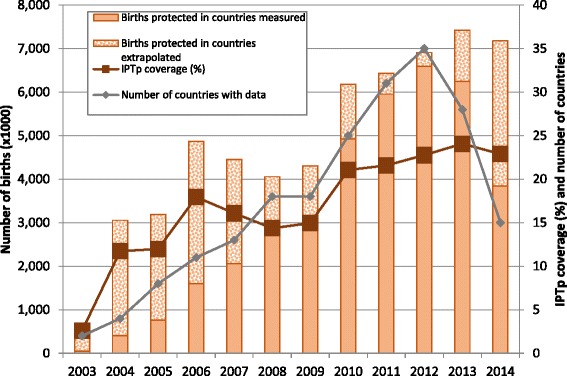
IPTp coverage in sub-Saharan Africa between 2003 and 2014

Current coverage estimates of IPTp and insecticide-treated nets (ITNs) in sub-Saharan Africa were presented by Anna Maria van Eijk (Liverpool School of Tropical Medicine, UK) for the period between 2003 and 2014 based on previously published data [1, 2], highlighting an alarming lack of IPTp scale-up. Although coverage for IPTp increased from less than 5 % in 2003 to above 20 % in 2010, no further progress has been made with average coverage rates that have since stagnated between 22 % and 24 %. Overall, IPTp coverage estimates remain far below global targets set by the RBM Partnership of 80 % by 2010, and 100 % (universal coverage) by 2015. Although six countries (The Gambia, Ghana, Malawi, Sao Tomé and Principe, Senegal and Zambia) reached the original 60 % coverage target for 2005, the combined estimate for all countries with an IPTp policy was 24 % in 2013 (Fig. 1). It is of particular concern that the latest estimates for 2014 indicate that coverage is falling in some countries. Progress for ITN coverage is comparatively better but still unacceptably low at 38 % overall. Production of ‘rolling coverage estimates’ are essential to track overall progress as well as to estimate the uptake and impact of the revised WHO recommendation for IPTp [3]. WHO updated the IPTp policy recommendation in September 2012, increasing the regimen from at least two doses of SP to the provision of a dose of IPTp-SP given at every ANC visit beginning as early as possible in the 2nd trimester throughout pregnancy and at least one month apart, ideally administered as directly observed therapy [3]. Measuring the impact of the policy update will require national household surveys to include data on two, three, and more than three doses of IPTp.

### Factors affecting antenatal care delivery of IPTp

Examples from observational studies in Mali and Kenya presented by Jayne Webster (LSHTM, UK) showed that IPTp delivered by direct observation at antenatal care (ANC) facilities was low and ineffective. Despite different operating contexts across countries in terms of IPTp policy on timing of doses and HIV prevalence, the same intermediate steps appeared to impede receiving any SP, and receiving SP by direct observation. SP stock-outs were not an issue when surveys were conducted. Interestingly, surveys revealed high variability across health facilities within the same district and highlighted the need to monitor and to intervene at the facility level to overcome barriers to IPTp, as well as to make distinctions between issues that are driven by individual providers and, separately, those related to facilities. Surveys employed mixed-methods to identify ineffective processes and their predictors for the delivery of IPTp with qualitative analysis offering deeper insights into certain practices as summarized in Table 1. These surveys enabled an in-depth assessment of the ANC processes that are required to deliver IPTp as well as to quantify the major barriers to its provision. Moreover, evidence from mixed-methods is needed to inform policymakers and programme managers on the most appropriate interventions that can improve the uptake of IPTp as well as other ANC interventions.

**Table 1:** Factors affecting the effectiveness of IPTp delivery through antenatal care platform: comparison between factors identified through quantitative and qualitative approaches [14]

Predictors of receiving any IPTp	Qualitative explanation
Education	None
Gestation	Misinterpretation of guidelines on the lower and upper limit of the gestational age at which SP could be given
Reason for attending ANC	If a pregnant woman is ill she first will be attended to for that illness and she may not receive routine ANC services
Having malaria symptoms	As above, illness usually dealt with first and IPTp should not be given concurrently with other anti-malarial treatment
Having the abdomen examined and palpated during ANC visit	None
Total amount of money spent	SP was sometimes sold at health facilities although it should be provided free of charge; women spending money were more likely to receive IPTp
None	Due to fear of SP side-effects when taken on an empty stomach, IPTp was not given during ANC visit nor given to be taken at home

**Fig. 2:**
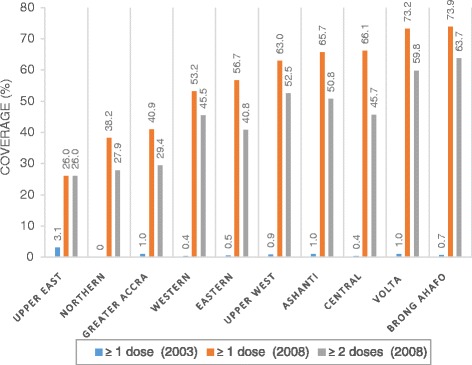
IPTp coverage across districts in Ghana in 2003 and 2008

Harry Tagbor (Kwame Nkrumah University of Science and Technology, Ghana) presented encouraging progress in IPTp uptake in Ghana between 2003 and 2008. National coverage was below 5 % in 2003 and rose to 44 % in 2008, although with marked variation between regions (see Fig. 2). A study from the rural district of Ashanti in 2009 found over 80 % of pregnant women to have received at least two doses of IPTp while 71 % of women made more than four ANC visits during their pregnancies. The number of ANC visits was significantly associated with greater IPTp dosing. This study further highlighted the importance of making every ANC visit count as an opportunity to deliver IPTp, and also the need to address bottlenecks at the local district and health facility levels.

The need for improved communication and messaging around the benefit of IPTp was illustrated by Matthew Chico (LSHTM, UK) from the findings of a qualitative investigation carried out in Tanzania in 2013 into the barriers to IPTp [4]. Knowledge of IPTp and an understanding of the difference between prevention and treatment of malaria among pregnant women in the study area was low. Many women reported that SP had undesirable side effects, including the misconception that IPTp negatively prolonged pregnancy, thereby increasing the risk to maternal health at delivery. It is possible that scientific evidence of IPTp protection against pre-term birth has been misinterpreted by pregnant women so that SP use in pregnancy is viewed as something undesirable or unsafe. Such beliefs and lack of awareness of malaria prevention interventions have potentially significant implications on the uptake of IPTp and should be addressed through appropriate behaviour change communication strategies. The same study showed strong demand in Tanzania from national policymakers, district-level healthcare providers and pregnant women at ANC facilities for holistic approaches that protect against MiP and curable sexually transmitted and reproductive tract infections in pregnancy.

### Cost-effectiveness of IPTp with SP

Findings from cost-effectiveness analyses of IPTp with SP (IPTp-SP) were presented by Elisa Sicuri (Barcelona Centre for International Health Research, Spain) who showed that two doses of IPTp-SP was highly cost-effective and that three or more doses of IPTp-SP was more cost-effective than two doses. The first analysis estimated the cost-effectiveness of IPTp-SP from a randomized placebo-controlled trial of IPTp-SP in the context of ITN use and moderate SP resistance in Mozambique [5]. This trial found that two doses of IPTp-SP against IPTp-placebo had a protective efficacy of 40 % (95 % Confidence Intervals [95 %CI]: 7.4; 61) for maternal clinical malaria and 61 % (95 % CI: 7.4; 83.8) for neonatal mortality. A threshold analysis was conducted to estimate cut-off points of the main variables beyond which IPTp-SP may no longer be cost-effective. Even assuming a reduction in SP efficacy to 15 % in clinical case management and a reduction in neonatal deaths averted from 11 to 4.5 per 1,000 women taking the prevention, IPTp-SP remained cost-effective. The study concluded that two doses of IPTp-SP is a highly cost-effective intervention both in terms of prevention of maternal clinical malaria and reduction in neonatal mortality in this context (Table 2). Furthermore, provision of IPTp with a more efficacious drug than SP, even if more expensive, may still remain a cost-effective public health measure to prevent malaria in pregnancy, particularly in HIV-infected pregnant women [6]. The second cost-effectiveness study was based on the meta-analysis of IPTp with three or more IPTp doses of SP versus two doses across a range of African settings [7]. The meta-analysis found that three or more doses of IPTp-SP was associated with increased birth weight and decreased risk of low birth weight, placental malaria and maternal parasitaemia compared to the standard two-dose regimen. Analysis showed that three or more doses of IPTp-SP were more cost-effective compared to two doses of IPTp-SP. This finding remained robust even if the underlying population level risk of low birth weight changes or if only HIV-negative women were considered. An update of this cost-effectiveness analysis has since been published [8]. However, the results might not be generalizable to areas with lower malarial transmission and/or with very high levels of malaria parasite resistance to SP [9].

**Table 2:** Cost-effectiveness of IPTp-SP

	Intervention costs/ DALYs averted in US$ for 2012	Intervention
Results on clinical malaria [5]	46.02	IPTp-SP 2 doses vs Placebo
Results on neonatal mortality [5]	1.20	IPTp-SP 2 doses vs Placebo
Combined analysis for clinical malaria and neonatal mortality [5]	1.13	IPTp-SP 2 doses vs Placebo
Combined analysis for clinical malaria, maternal anaemia and low birth weight [8]	7.28	IPTp-SP 3+ doses vs IPTp-SP 2 doses

### Strengthening health systems and the antenatal care platform

Kate Mitchell (Harvard School of Public Health/Maternal Health Task Force, USA) described the current shift in the delivery of ANC. The ANC platform is a key point of entry into the formal health system and presents a unique opportunity to reach the majority of pregnant women. Although ANC attendance is relatively high, even in low income countries, further efforts need to focus on the quality of care and service delivery. A recent study has drawn attention to the enormous gap between coverage as measured by attendance and coverage with specific ANC interventions [10]. Several recent studies stressed other challenges with and barriers to accessing ANC such as hidden costs (transportation, time off work, purchase of drugs on the private market when there were stock outs at health facilities); cultural factors influencing timing of pregnancy disclosure and healthcare workers negative attitudes exhibited towards women who present at ANC facilities [11]. Findings from a recent secondary analysis of the original focused ANC trials showed an increase in perinatal mortality which is of great concern and needs to be further researched [12]. Although the original WHO-sponsored focused ANC trial did not include sites from sub-Saharan Africa, the WHO is working with numerous partners to revise ANC guidelines through technical consultations that have included mapping of ANC guidelines worldwide and defining a new women-centred approach to ANC delivery. Revision of ANC guidelines will need to consider ways to increase healthcare worker adherence to delivery of IPTp and to yield better uptake among women. This is a critical time to revisit antenatal care policy and consider the following issues: harmonization of policies, guidelines, and recommendations; draw on lessons learned from slow IPTp uptake; review the role of community health workers to increase uptake of both ANC and IPTp; ensure a women-centred approach is adopted; tap into funding streams which support integration of maternal and newborn health.

Beyond the ANC platform, Elaine Roman (JHPEIGO, USA) discussed the need to target shortcomings in the following components of the health system to improve coverage of IPTp and key interventions: better integration of policies and guidelines between Reproductive Health and National Control Malaria Programmes; focus on healthcare worker capacity development both pre- and in-service training; quality assurance through support supervision; community engagement for early ANC attendance and IPTp uptake; ensuring availability of commodities at ANC facilities including SP for IPTp; monitoring and evaluation that makes use of facility-level data for decision making; securing adequate financial support.

Moving forward, strengthening ANC and components of the health system that are based on specific country contexts will be necessary to scale up IPTp coverage and have lasting health benefit for both mother and newborn.

A summary of the key issues and recommendations for scale-up of IPTp-SP discussed by seminar participants following the presentations is provided below. A comprehensive list of recommended actions by stakeholder group is presented in a companion paper [15].

1. What are we calling malaria-endemic countries to do?
  1.1. Healthcare workers play a critical role in making IPTp attractive to pregnant women. There is a need to address negative healthcare-worker attitudes and behavioural issues such as abuse and disrespect as commonly reported by ANC clients. The importance of healthcare worker welfare must be considered alongside issues such as commodities and infrastructure needs. In low- and middle-income countries health facilities are generally under-staffed and healthcare workers overburdened. Motivated healthcare workers are more likely to improve delivery of IPTp and uptake by ANC clients.
  1.2. RBM developed guidelines for behaviour change communication (BCC) [13] which are useful for increasing demand for and acceptance of IPTp. Such a communication programme should not only address knowledge gaps but also encourage self-advocacy and address perceived risks of malaria infection and preventive treatment. A successful BCC strategy would result in pregnant women demanding to be protected from malaria.
  1.3. Pre-service training as well as continuous medical training focused on malaria in pregnancy and IPTp can play a critical role in the scale up of IPTp. This is currently done for Prevention of Mother to Child Transmission of HIV (PMTCT) but not uniformly for malaria in pregnancy.
  1.4. The distribution of simplified guidelines on the IPTp policy signed by local and national authorities has proven effective at scaling up IPTp in western Kenya.
  1.5. There is a need to ensure greater integration between different public health programmes, not only Reproductive Health and Malaria, but also TB and HIV so as to strike the right balance across all activities. PMTCT is an important part of focused ANC and has received disproportionate attention, taking over most of the ANC process which can overshadow other interventions. It is also important to recognize that HIV-positive women on prophylactic co-trimoxazole are not eligible for IPTp with SP, and this group represents up to a third of all pregnant women attending ANC facilities in certain regions. HIV positive pregnant women are more susceptible to malaria than their HIV negative counterparts and yet they receive sub-optimal malaria prevention with co-trimoxazole. Non-sulpha based alternative therapies are needed that can be taken concurrently with co-trimoxazole.
  1.6. Given the evidence that high-dose folic acid (5 mg or above) counteracts SP efficacy, countries need to ensure low-dose folic acid (0.4 mg) is available for ANC use.
  1.7. Community engagement and involvement have demonstrated potential to raise awareness of and to demystify challenges associated with ANC and IPTp, and to build momentum for early ANC attendance and IPTp uptake. Therefore, there is a need for active community involvement in mobilizing and educating pregnant women about the benefits of early ANC attendance and use of SP for prevention of malaria during pregnancy. Active community participation remains one of the central tenets of primary health care, and is essential to building strong health systems and to meeting the healthcare needs of pregnant women.
2. What are we calling the Global Fund to do?
  2.1. Although most governments in sub-Saharan Africa have stated policies to provide ANC services at no cost to users, the reality is that health facilities usually function on some form of cost-recovery basis. Therefore, pregnant women are often expected to pay for ANC registration and/or laboratory tests. To ensure services are indeed free, public health budgets should cover the cost of commodities as well as incidental expenses, such as the cost of transport from central depots to health facilities.
  2.2. Each year during the Global Fund proposal preparation, countries are faced with tough decisions about which components to include in budgets. For various reasons, the cost of procuring SP and delivering IPTp is often considered an in-kind expense that is to be covered by applicants. However, there is often no process in place to ensure these expenses are actually met at the government level. Countries should be encouraged to budget these costs in their Global Fund applications. If these costs are not included, then there should be a strong agreement in place at the national level stating that countries will procure SP and support integration of Reproductive Health and Malaria programmes.
3. What are we calling the researchers to do?
  3.1. Develop, test and evaluate successful BCC strategies which result in pregnant women demanding to be protected from malaria infection.
  3.2. Evaluate the evidence on the effectiveness and drawbacks of community distribution of IPTp.
  3.3. Improve dissemination of research and lessons learned from existing evidence.
4. What are we calling the pharmaceutical industry to do?
  4.1. In some settings, the procurement of SP has become a considerable challenge; the pharmaceutical industry/manufacturers should be encouraged to register SP in countries that have IPTp policies.
  4.2. Most countries are still providing high-dose folic acid which has been shown to decrease SP efficacy. The pharmaceutical industry needs to align with ANC programmes to manufacture and to register low-dose folic acid.
  4.3. To investigate anti-malarial drugs that could replace SP and be given safely in the first trimester of pregnancy.
5. What are we calling private health care providers to do?
  5.1. The importance of private healthcare providers is often overlooked. However, their importance in health service provision is growing in low and middle-income countries (up to 50 % in certain areas). Private healthcare providers, including facilities operated by non-governmental organizations and faith-based organizations, should be included in IPTp training, supervision and supported with an uninterrupted supply of quality SP.

## Additional files

Additional file 1:
**Call to Action to Scale-up IPTp Coverage - Seminar Agenda.**
Additional file 2:
**List of participants at the Call to Action for the Scale-up IPTp seminar during the 63rd Annual Meeting of the American Society of Tropical Medicine and Hygiene in New Orleans, USA.**

## Abbreviations

ANC: Antenatal care
BCC: Behaviour change communication
GMP: Global Malaria Programme
IPTp: Intermittent preventive treatment of malaria in pregnancy
ITNs: Insecticide-treated nets
LSHTM: London School of Hygiene and Tropical Medicine
MiP: Malaria in Pregnancy
MiPc: the Malaria in Pregnancy Consortium
PMTCT: Prevention of Mother to Child Transmission of HIV
RBM: the Roll Back Malaria
SP: Sulphadoxine-pyrimethamine
WHO: World Health Organization
