# Supplementary figures and images for: Global Call to Action to scale-up coverage of intermittent preventive treatment of malaria in pregnancy: seminar report
Source: Malar J. 2015 May 18;14:206. doi: 10.1186/s12936-015-0730-3 (PMC4446906; doi:10.1186/s12936-015-0730-3)

# Additional file 1: Call to Action to Scale-up IPTp Coverage- Seminar Agenda


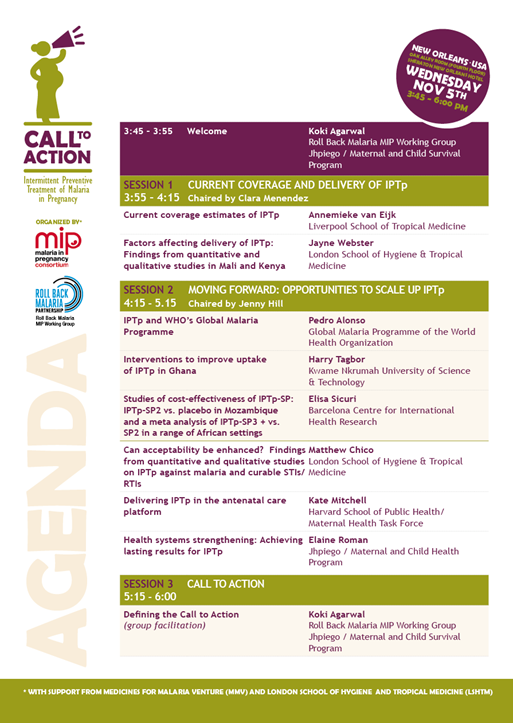

Supplement: Additional file 1: — Call to Action to Scale-up IPTp Coverage - Seminar Agenda. [file 12936_2015_730_MOESM1_ESM.docx]
