# Supplementary material for: Global Call to Action to scale-up coverage of intermittent preventive treatment of malaria in pregnancy: seminar report
Source: Malar J. 2015 May 18;14:206. doi: 10.1186/s12936-015-0730-3 (PMC4446906; doi:10.1186/s12936-015-0730-3)
Supplement: Additional file 2: — List of participants at the Call to Action for the Scale-up IPTp seminar during the 63rd Annual Meeting of the American Society of Tropical Medicine and Hygiene in New Orleans, USA. [file 12936_2015_730_MOESM2_ESM.docx]

# Additional file 2: List of participants at the Call to Action for the Scale-up IPTp seminar held during the 63rd Annual Meeting of the American Society of Tropical Medicine and Hygiene (ASTMH), New Orleans, USA, 5th of November 2015

Agarwal Koki RBM MiP WG Co-Chair / JHPIEGO; Alonso Pedro Global Malaria Program/WHO HQ, Geneva; Aspinall Adam MMV; Balu Ebenezer Malaria Consortium; Banek Kristin LSHTM; Bardají Azucena Barcelona Institute for Global Health; Belay Kassahua USAID/PMI; Bosman Andrea WHO HQ Geneva; Brandling-Bennett David Gates Foundation ; Briand Valerie Institut de Recherche pour le Développement ; Bruce Jane LSHTM; Chico Matthew LSHTM; Claite Manuela LSHTM ; Cot Michel IRD; D'Alessandro Umberto MRC the Gambia ; de Gier Nicole Abt. Associates ; Dellicour Stephanie MiP Consortium/LSTM ; Desai Meghna KEMRI/CDC ; Duparc Stephan MMV ; Finley Michael ExxonMobil; Florey Lia ICF International-DHS Program; Greenwood Brian LSHTM; Guerin Philippe WWARN; Gutman Julie CDC; Hamel Mary CDC; Hill Jenny MiP Consortium/LSTM; Hornston Sureyya USAID; Inyang Uwem USAID Nigeria; Juma Elizabeth KEMRI; Kafuko Jessica USAID/PMI; Kariuki Muthoni JHPIEGO; Kayentao Kassoum University of Bamako; Kovacs Stephanie University of Washington; Kuepfer Irene LSHTM; Linn Anne Rutgers University; Lynch Matt CCP; Madanitsa Mwayi University of Malawi; Magnussen Pascal University of Copenhagen; Maheu-Giroux Mathieu Harvard SPH ; Majeres Lugand Maud MMV ; Massougbodji Achille CERPAGE; Menendez Clara Barcelona Institute for Global Health; Mitchel Kate MHTF; Momoh Veronica PMI/MAPS Project; Mosha Jaqueline NIMR / Tanzania; Moss Bill JHU; Munthali John JHPIEGO; Mwapasa Victor College of Medicine (Malawi); Nahlen Bernard PMI; Nankabinoa Joaniter Malaria Consortium; Ngindu Augustine JHPIEGO; Ojo Akinyeye Abiodun FHI 360 Nigeria; Orji Bright JHPIEGO; Ouma Peter KEMRI; Paintain Lucy LSHTM; Phillips Howard Penny LSTM; Qorro Grace JHPIEGO; Reynolds Alison Imperial College; Ricotta Emily JHU; Riley Christina CDC; Rogerson Stephen University of Melbourne; Roman Elaine JHPIEGO ; Sané Binetou MMV ; Siame Mwiche Malaria Institute at Macha; Sicuri Elisa CRESIB; Slutsker Larry CDC; Steketee Rick PATH; Tagbor Harry KNUST; Ter Kuile Feiko MiP Consortium/LSTM; Thuma Phil Malaria Institute at Macha; Toso Michel JHU.CCP; UDOM Boi-Betty RBM Secretariat; Unger Holger University of Melbourne; Valea Innocent IRSS/Centre Muraz; van Eijk Annemieke LSTM; Walker Patrick Imperial College; Webster Jayne LSHTM; Wolf Kate JSI; Youll Susan PMI; Zigirumugabe Sixte PMI
